# Human Umbilical Cord Blood Serum–derived α-Secretase: Functional Testing in Alzheimer’s Disease Mouse Models

**DOI:** 10.1177/0963689718759473

**Published:** 2018-03-21

**Authors:** Ahsan Habib, Huayan Hou, Takashi Mori, Jun Tian, Jin Zeng, Shengnuo Fan, Brian Giunta, Paul R. Sanberg, Darrell Sawmiller, Jun Tan

**Affiliations:** 1 Department of Psychiatry and Behavioral Neurosciences, Morsani College of Medicine, University of South Florida, Tampa, FL, USA; 2 Departments of Biomedical Sciences and Pathology, Saitama Medical Center and Saitama Medical University, Kawagoe, Saitama, Japan.; 3 Department of Neurosurgery and Brain Repair, Center for Aging and Brain Repair, Morsani College of Medicine, University of South Florida, Tampa, FL, USA

**Keywords:** Alzheimer’s disease, amyloid-β, cord blood serum, human umbilical cord blood cell, soluble amyloid precursor protein α, tau

## Abstract

Alzheimer’s disease (AD) is an age-related disorder that affects cognition. Our previous studies showed that the neuroprotective fragment of amyloid procurer protein (APP) metabolite, soluble APPα (sAPPα), interferes with β-site APP-cleaving enzyme 1 (BACE1, β-secretase) cleavage and reduces amyloid-β (Aβ) generation. In an attempt to identify approaches to restore sAPPα levels, we found that human cord blood serum (CBS) significantly promotes sAPPα production compared with adult blood serum (ABS) and aged blood serum (AgBS) in Chinese hamster ovary cells stably expressing wild-type human APP. Interestingly, CBS selectively mediated the α-secretase cleavage of human neuron-specific recombinant APP_695_ in a cell-free system independent of tumor necrosis factor-α converting enzyme (TACE; a disintegrin and metalloproteinase domain-containing protein 17 [ADAM17]) and ADAM. Subsequently, using 3-step chromatographic separation techniques (i.e., diethylaminoethanol, size-exclusion, and ion-exchange chromatography), we purified and ultimately identified a CBS-specific fraction with enhanced α-secretase catalytic activity (termed αCBSF) and found that αCBSF has more than 3,000-fold increased α-secretase catalytic activity compared with the original pooled CBS. Furthermore, intracerebroventricular injection of αCBSF markedly increased cerebral sAPPα levels together with significant decreases in cerebral Aβ production and abnormal tau (Thr^231^) phosphorylation compared with the AgBS fraction with enhanced α-secretase activity (AgBSF) treatment in triple transgenic Alzheimer’s disease (3xTg-AD) mice. Moreover, AgBSF administered intraperitoneally to transgenic mice with five familial Alzheimer’s disease mutations (5XFAD) *via* an osmotic mini pump for 6 weeks (wk) ameliorated β-amyloid plaques and reversed cognitive impairment measures. Together, our results propose the necessity for further study aimed at identification and characterization of α-secretase in CBS for novel and effective AD therapy.

## Introduction

The neuropathological hallmarks of Alzheimer’s disease (AD) that differentiate it from other types of dementia include extracellular β-amyloid plaques composed largely of amyloid-β (Aβ) peptides^1^ and intracellular neurofibrillary tangles (NFTs) composed of the hyperphosphorylated microtubule-associated protein tau^2^. Successive cleavage of amyloid procurer protein (APP) by β- and γ-secretases produces Aβ peptides of variable length (Aβ_x-40, 42_), soluble APPβ, membrane-bound β-C-terminal fragment (β-CTF, C99), and APP intracellular cytoplasmic/C-terminal domain (AICD)^3^. The Aβ peptide fragments, which accumulate as plaques in the brain, induce neuroinflammation, synaptic dysfunction, and neuronal cell death that affects cognitive function^4^. In contrast, most of the APP is cleaved by α- and γ-secretases that not only preclude Aβ generation but also produce a secreted soluble APPα (sAPPα), membrane-bound α-CTF (α-CTF, C83), P3 peptide, and AICD^5^. Overall, sAPPα has been shown to be involved in numerous physiological functions in the brain, which appear to be interrupted in AD. Several studies have shown that neurotrophic fragment sAPPα not only prevents Aβ generation^6^ and tau phosphorylation^7^ but is also known to be a neuroprotective APP metabolite including but not limited to proliferation, neurite outgrowth, and long-term potentiation^8–10^. Thus, we hypothesized that therapeutic interventions or approaches that have potential to produce sAPPα markedly could improve AD pathology and cognitive function.

Several studies have shown that human umbilical cord blood cells (HUCBCs) have therapeutic potential in numerous age-related neuroinflammatory conditions including AD. In line with those studies, we showed that single as well as multiple low-dose infusion of HUCBC significantly reduced amyloidogenic APP processing, Aβ and β-amyloid plaque accumulation, glial neuroinflammation, and cognitive impairments in preclinical AD mouse models^11,12^. Additionally, HUCBC treatment changed microglial phenotypes from pro-inflammatory to anti-inflammatory, increased microglial Aβ phagocytosis, increased anti-inflammatory cytokines in the brain (i.e., interleukin-10, transforming growth factor β1, and nerve growth factor), and reduced CD40 receptor-CD40 ligand (CD40-CD40L) interaction that is important for Aβ-induced pro-inflammatory microglial activation^13^. To identify the specific HUCBC responsible for this neuroprotective effect, we found that cord blood–derived monocyte reduces β-amyloid pathology and improves cognition with much more effectively than monocyte-deficient cord blood in AD mouse model^13^. In line with the findings of above studies, several recent reports underscored the role of young blood and/or plasma in aging and age-associated neurodegenerative conditions. Among those, Wyss-Coray and other labs have reported that exposing old mice to a young systemic environment by parabiosis increased synaptic plasticity, improved pathology, and behavioral recovery such as contextual fear conditioning and spatial learning in old mice. More interestingly, they also found that it is not the blood cells rather the soluble factors that are getting into the mice brain. They pooled plasma from young mice as well as from young human and injected into old mice, which successfully rejuvenated old mice brain structure and cognition tested by Barnes maze memory test^14–18^. In a follow-up study, they showed that human cord blood plasma (CBP) as well as plasma enriched in tissue inhibitor of metalloproteinases 2 improves synaptic plasticity and hippocampal-dependent cognitive function in old mice^19^.

Based on our preliminary laboratory findings, we hypothesized that human cord blood serum (CBS) possesses novel APP-specific α-secretase-like enzyme, reflected by marked increase in sAPPα level. As CBS contains many different small molecules, growth factors, proteins, inhibitors, hormones, enzymes, and other unknown substances, we also hypothesized that infusion of characterized CBS fraction will ameliorate AD-like pathology and cognitive impairments in mouse models. Here, we show that CBS markedly enhanced the level of sAPPα in Chinese hamster ovary (CHO) cells stably expressing wild-type human APP (CHO/APPwt cells) as well as mediated α-secretase cleavage of human neuron-specific APP_695_ (fAPP_695_) in a cell-free system, which effects are not seen with normal adult or aged blood serum (ABS or AgBS). Additionally, we have been successfully able to characterize a CBS fraction with enhanced α-secretase-like catalytic activity (refer to αCBSF) using sequential diethylaminoethanol (DEAE)-affinity column, size-exclusion, and anion-exchange chromatographic fractionation processes. Moreover, we found that αCBSF infusion increased sAPPα levels, decreased Aβ production/β-amyloid plaque formation, prevent neuronal loss and abnormal tau (Thr^231^) phosphorylation in the cortex, and improved cognitive function in Alzheimer’s mouse models. Our findings indicate that αCBSF holds immense therapeutic potential for treatment of AD.

## Materials and Methods

### Reagents and Antibodies

CBS was obtained from Lee Biosolutions (St. Louis, MO, USA, and human umbilical CBP was obtained from STEMCELL Technologies Inc. (Vancouver, British Columbia, Canada). Human cord blood is aspirated from the umbilical cord vein into a cord blood collection bag containing citrate–phosphate–dextrose as an anticoagulant. Individual lot of CBP is prepared from a single cord blood sample. Three to five different lots of CBP samples were pooled in as “pooled CBP.” CBP is separated from umbilical cord blood centrifugation at 3,500 rpm for 5 to 10 min. CBP is aliquoted and frozen at −20 °C first and then transferred to a −80 °C freezer after 24 h at 4 °C. There is no placement into −80 °C for a snap freeze. Frozen CBP is not heat inactivated. No analysis was performed to determine the number of platelets in each sample; therefore, the plasma cannot be specifically characterized as “low-platelet” or “platelet-poor” plasma. Frozen CBP samples were thawed in a 37 °C water bath before being used in experiment. CBS was collected from umbilical cord blood; and it is the blood that remains in the placenta and in the attached umbilical cord after the cord has been detached from the newborn at the time of childbirth. CBS is separated from umbilical cord blood by allowing it to clot for 5 to 10 h in red top tubes with no anticoagulation followed by centrifugation at 3,500 rpm for 5 to 10 min at 4 °C. CBS sample was passed through a filter membrane with a pore size of 0.22 µm. Individual CBS was prepared from a single sample. More than 10 serum samples of CBS were pooled in as “pooled CBS.” Normal human adult blood serum (ABS, 25 to 30 years old) and AgBS (>75 years old) as well as their plasma (ABP and AgBP, respectively) were obtained from Florida Blood Services (Tampa, FL, USA). Antibodies include specific anti-sAPPα monoclonal antibody (2B3; IBL, Minneapolis, MN, USA, Cat# 11088 RRID: AB_494690), anti-APP C-terminal polyclonal antibody (pAb751/770; EMD Millipore, La Jolla, CA, USA, Cat# 171610, RRID: AB_211444), anti-APP N-terminal monoclonal antibody (22C11; Merck Millipore, Billerica, MA, USA, Cat# MAB348B, RRID: AB_11204540), anti-N-terminal Aβ monoclonal antibody (6E10; Covance Research Products, Emeryville, CA, USA), anti-Aβ_16–26_ monoclonal antibody (4G8; Covance Research Products, Cat# SIG-39200, RRID: AB_10175149), anti-phospho-tau antibody (Thr^231^; Merck Millipore, Billerica, MA, USA, Cat# AB9668, RRID: AB_570891), anti-DDK-tagged antibody (Cell Signaling Technology, Danvers, MA, USA, Cat# 2908, RRID: AB_1905079), anti-NeuN antibody (Merck Millipore, Billerica, MA, USA, Cat# ABN90, RRID: AB_11205592), and anti-β-actin monoclonal antibody (Sigma-Aldrich, St. Louis, MO, USA, Cat# A5316, RRID: AB_476743). Human recombinant full-length APP_695_ (fAPP, 100 ng) tagged with C-terminal MYC/DDK was purchased from OriGene Technologies, Inc. (Rockville, MD, USA).

### Cell Culture

CHO cell line with stable expression of human wild-type APP (CHO/APPwt) was a generous gift from Drs. Stefanie Hahn and Sascha Weggen (University of Heinrich Heine, Düsseldorf, Germany). At the beginning of the experiment, CHO/APPwt cells were genotyped and confirmed the genetic makeup. The cells were cultured in Dulbecco’s modified Eagle’s medium (DMEM) with 10% fetal bovine serum (FBS), 1 mM sodium pyruvate, and 100 U/mL of penicillin/streptomycin. For treatment, the cells were plated in a 24-well plate at 2 × 10^5^ cells/well for overnight incubation, washed and treated with CBS (0% to 10%), inact CBS (5%), ABS (0% to 10%), AgBS (0% to 10%), or αCBSF (0% to 1%) in DMEM. After treatment, supernatants were collected and the cells were washed with ice-cold phosphate-buffered saline (PBS) 3X and lysed with cell lysis buffer (20 mM Tris, pH 7.5, 150 mM NaCl, 1 mM EDTA, 1 mM EGTA, 1% v/v Triton X-100, 2.5 mM sodium pyrophosphate, 1 mM β-glycerophosphate, 1 mM Na_3_VO4, 1 µg/mL leupeptin, and 1 mM phenylmethane sulfonyl fluoride; Cell Signaling Technology, Danvers, MA, USA). Both cell supernatants and lysates were used for sAPPα analysis by ELISA. For immunoprecipitation, 100 ng of fAPP_695_ was incubated with αCBSF at 37 °C for 1 h, and then the sAPPα/αCBSF-derived immune complex was immunoprecipitated using 2B3 sAPPα-specific antibody or anti-DDK antibody. The supernatants were then collected and used for treatment of CHO/APPwt cells.

### Cell-free α-secretase Assay

In order to determine the α-secretase activity of CBS reflected by sAPPα level, human recombinant fAPP_695_ tagged with C-terminal MYC/DDK (100 ng; OriGen Biomedical, Austin, TX, USA) was incubated with CBS, heat inactivated CBS (inact CBS, 56 °C for 30 min), AgBS, FBS, or αCBSF at 37 °C for 5 h in the presence or absence of protease inhibitor (PI) cocktail (1X; Sigma-Aldrich, St. Louis, MO, USA), tumor necrosis factor-α (TNFα) converting enzyme (TACE) inhibitor (TAPI-0) (1 µM; Abcam, Cambridge, MA, USA), or a disintegrin and metalloproteinase domain–containing protein (ADAM) inhibitor (GM6001, 1 µM; Sigma-Aldrich, St. Louis, MO, USA). The reaction mixtures were then subjected to Western blot (WB) analysis for APP α-secretase processing. In addition, TACE and ADAM10 cleavage activity of αCBSF was determined using TACE (AnaSpec, Fremont, CA, USA) and ADAM10 cleavage activity kits (AnaSpec, Fremont, CA, USA), according to the manufacturer’s instructions.

### CBS Fractionation

Next, in order to purify and characterize the α-secretase in CBS or AgBS, the Econo-Pac Serum IgG Purification Kit, and 10DG columns (Bio-Rad, Philadelphia, PA, USA) were initially employed to remove highly abundant IgG and salts. The desalted serum was applied to DEAE Affi-Gel Blue columns, and residual IgG was eluted according to the instructions. Then, 20 additional protein fractions were collected by eluting with an ionic strength gradient of NaCl buffer ranging from 0.1 M to 2.0 M. The remaining proteins on the column were eluted by the regeneration buffer included in the kit and collected as the regeneration fraction. The 0.8 M NaCl-eluted protein fractions were combined together and sent to Moffitt Cancer Center Protein Purification Core (Tampa, Fl, USA) for further separation by size-exclusion chromatography, employing analytic Superdex 200 columns and eluting with PBS, and ion-exchange chromatography, employing Q-Sepharose columns and eluting with 500 mM NaCl, 50 mM Tris, pH 7.6. The final enzyme containing fractions was exchanged to PBS by Ultracel-10 membranes (10 kDa, Merck Millipore, Billerica, MA, USA) for further experimentation and referred to as αCBSF or AgBSF.

### Animal Models

Both 5XFAD (MMRC Stock No: 34840-JAX; RRID IMSR_JAX: 006554) and 3xTg-AD (MMRC Stock No: 34830-JAX) mice of male and female were purchased from Jackson Laboratory (Bar Harbor, Maine, USA). In this preclinical study to investigate whether CBS fractionation changes AD-like pathology and associated behavioral deficits, 5-month-old 5XFAD mice were used that harbor 5 mutations (APP KM670/671NL [Swedish], APP I716V [Florida], APP V717I [London], PSEN1 M146L, and PSEN1 L286V)^20^ and rapidly develop AD-like pathology including accumulation of high levels of extracellular β-amyloid plaques, neurodegeneration, and behavioral impairments. In order to investigate whether CBS fractionation administration changes both Aβ and tau phosphorylation, 3xTg-AD mice which harbor presenilin-1 (PS1/M146V), APP (KM670/671NL), and tau (P301L) mutants were used. These mice progressively develop β-amyloid and NFT pathology, which potentially synergize to accelerate neurodegeneration at the age of 6 months (6 mo)^21^. At the beginning of the experiment, all mice were confirmed as mutant by polymerase chain reaction. One male and 4 female mice were housed in a single cage separately. All animal experiments were performed in accordance with the guidelines of the National Institutes of Health and were approved by University of South Florida (USF) Institutional Animal Care and Use Committee (IACUC reference number: IS00000438). Transgenic mice used for aging studies may exhibit signs such as ruffled hair coat, hair loss, excessive weight gain, and/or loss. When one of these signs observed, mice were monitored more closely and weighed twice weekly. Mice exhibited multiple clinical signs or showing >20% weight loss were excluded from the study. As per our previous practice, if a mouse appears overtly sick or in pain as indicated by ruffled, matted, or dull hair; hunched back or head pressing; failure to move about the cage; failure to respond to stimuli, rapid, shallow, labored breathing, twitching or trembling; or failure to experience seizure, a veterinarian was consulted in order to ensure timely intervention and treatment or removal from the study. All mice were maintained on a 12-h light/12-h dark cycle at ambient temperature and humidity and housed in the Morsani College of Medicine Animal Facility at the USF with ad libitum access to food and water.

### Stereotaxic Intracerebroventricular (i.c.v.) Injection

In order to determine whether αCBS fraction could modify Aβ and tau pathology, cohorts of 17 (*n* = 17, 9♂/8♀) triple transgenic 3xTg-AD mice were arbitrary anesthetized with isoflurane (2% to 3% induction, 1% maintenance). After reflexes were checked to ensure that mice were unconscious, they were positioned on a stereotaxic frame (Stoelting’s Lab Standard™, Wood Dale, IL, USA) with ear bars positioned and jaws fixed to a biting plate. The axis coordinates were taken from a mouse brain atlas, and the needle of a Hamilton microsyringe was implanted into the left lateral ventricle delimited from the stereotaxic coordinates (coordinates relative to bregma: −0.6 mm anterior/posterior, +1.2 mm medial/lateral, and −3.0 mm dorsal/ventral) using the stereotaxic device. αCBSF (0.5 µg/mouse, *n* = 6, 3♂/3♀), AgBSF (0.5 µg/mouse, *n* = 6, 3♂/3♀), and PBS (1 µL/mouse, *n* = 5, 3♂/2♀) were administered at 1 μL/min. After administration, the syringe was removed slowly to prevent bleeding and further brain damage. The lesions were closed with 1 to 2 staples and observed until anesthesia had cleared. Seventy-two hour after the i.c.v. injections, animals were killed with isoflurane, then transcardially perfused with ice-cold PBS, and brains were harvested for biochemical, histochemical, and immunohistochemical analyses.

### Intraperitoneal (i.p.) Administration With an Osmotic Mini Pump

Mice were labeled using tail tattooing by veterinarian who was blinded about the entire experiment. In order to determine whether CBS fractionation changes AD-like pathology and associated behavioral deficits, cohort of even-number labeled 5XFAD mice was randomly assigned to 2 experimental groups of 6 mice each, receiving αCBSF (*n* = 6, 3♂/3♀) or AgBSF (*n* = 6, 3♂/3♀) treatment by an Alzet® osmotic mini pump (Alzet 2004, DURECT Corporation, Cupertino, CA, USA). A third group of wild-type (WT) control mice received αCBSF (*n* = 6) through the same administration route. Mice were briefly anesthetized with isoflurane as described previously, an area of the abdomen was shaved, a 1-cm abdominal incision was made and an Alzet® osmotic mini pump was filled with 100 μL of CBSF, or AgBSF was implanted i.p.. The pump delivered these fractionated sera at a constant rate of 0.15 µL/h for 6 wk, yielding a treatment dose of 1 mg/kg/day or 30 μg/mouse/day. At the end of 4- to 5-weeks (wk) treatment (6 mo of age), cognitive evaluations were conducted in these mice with our established behavioral battery. After 6 wk treatment, mice were killed with isoflurane, then transcardially perfused with ice-cold PBS, and brains were removed to assess β-amyloid plaque pathology.

### Behavioral Assessments

#### Novel object recognition test

Novel object recognition is based on the spontaneous tendency of a mouse to explore a new object compared with an old object. At first, during the habituation phase (day 1), each mouse was acclimatized with the testing apparatus box for 10 min. Next, during the training day (day 2), each mouse was familiarized with 2 similar objects (4 cm × 4 cm × 4 cm) for 10 min. Finally, during the testing day (day 3), one of the objects was replaced with a new object and tested for 10 min. The amount of time spent exploring the new and old objects during the test phase was quantified by video tracking (ANY-Maze; Stoelting’s Lab Standard™, Wood Dale, IL, USA) and provides an index of recognition memory. Discrimination index was calculated as the frequency of exploration of new *versus* original objects.

#### Y-maze test

Y-maze test was performed as described previously^22^. This task was used to assess basic mnemonic processing by spontaneous percentage alternation and exploratory activity of mice placed into a black Y-maze. The arms of this maze were 21-cm long and 4-cm wide with 40-cm high walls. Each mouse was placed in one of the arms and allowed one 5-min trial of free exploration of the 3 alleys in the maze. The numbers of total arm choices and sequence of arm choices were recorded.

### Histochemical and Immunohistochemical Analyses

Mice were euthanized with isoflurane and then transcardially perfused with ice-cold PBS. Brains were rapidly isolated, and one hemisphere was frozen immediately in liquid nitrogen and stored at −80 °C. For molecular analysis, brain hemispheres were sonicated in radioimmunoprecipitation assay buffer (Cell Signaling Technology, Danvers, MA, USA), centrifuged at 14,000 rpm for 1 h at 4 °C, and supernatants were isolated for WB analyses. The other hemisphere was placed in 4% paraformaldehyde for cryostat sectioning. The 25-μm free-floating coronal sections were collected and stored in PBS with 100 mM sodium azide at 4 °C. Immunohistochemical staining was performed using various primary antibodies in conjunction with the VECTASTAIN Elite ABC kit (Vector Laboratories, Burlingame, CA, USA) coupled with diaminobenzidine substrate. A set of sections without adding primary antibody were used as negative staining control. Sections were also stained with Congo red dye and Thioflavin-S fluorescence dye for detecting fibrillary Aβ species as described previously^23,24^. Images of five 25-μm sections (150 μm apart) through hippocampus and neocortex were captured, and a threshold optical density was obtained that discriminated staining from background. Data are reported as percentage of immunolabeled area captured (positive pixels divided by total pixels captured). Quantitative image analysis was performed by a single examiner (T.M.) blinded to sample identities.

### WB Analysis and ELISA

WB analyses and quantification were performed as previously described^25^. Briefly, the proteins from the cell-free suspensions, cell lysates, and homogenized tissue were electrophoretically separated using 10% bicine/Tris gel (8 M urea) for proteins less than 5 kDa or 10% Tris/sodium dodecyl sulfate (SDS) gels for larger proteins. Electrophoresed proteins were transferred to nitrocellulose membranes (Bio-Rad), washed, and blocked for 1 h at room temperature in Tris-buffered saline containing 5% (w/v) nonfat dry milk (TBS/NFDM). After blocking, membranes were hybridized overnight with various primary antibodies, washed, and incubated for 1 h with the appropriate horseradish peroxidase-conjugated secondary antibody in TBS/NFDM. Blots were developed using the luminol reagent (Thermo Fisher Scientific, Waltham, MA, USA). The sAPPα ELISA (IBL) was performed according to manufacturer’s instruction.

### Statistical Analysis

Comparison between 2 groups were performed by Student’s *t*-test analysis. For more than 2 groups, one-way analysis of variance followed by Least Significance Difference (LSD) post hoc analysis was used to compare each other for statistical significance. The α was set at *p* < 0.05 for all analyses. The significance level of *P* value was set at < 0.05 for all analyses. All the mice experiment were repeated 3 times in parallel to attain the above significant difference. Data are expressed as mean ± standard error of the mean. The statistical package for the social sciences released by IBM SPSS Version 23.0 (IBM, Armonk, NY, USA) was used for all data analyses.

## Results

### CBS Dose-Dependently Promotes α-cleavage in CHO/APPwt Cells

**Fig. 1.**
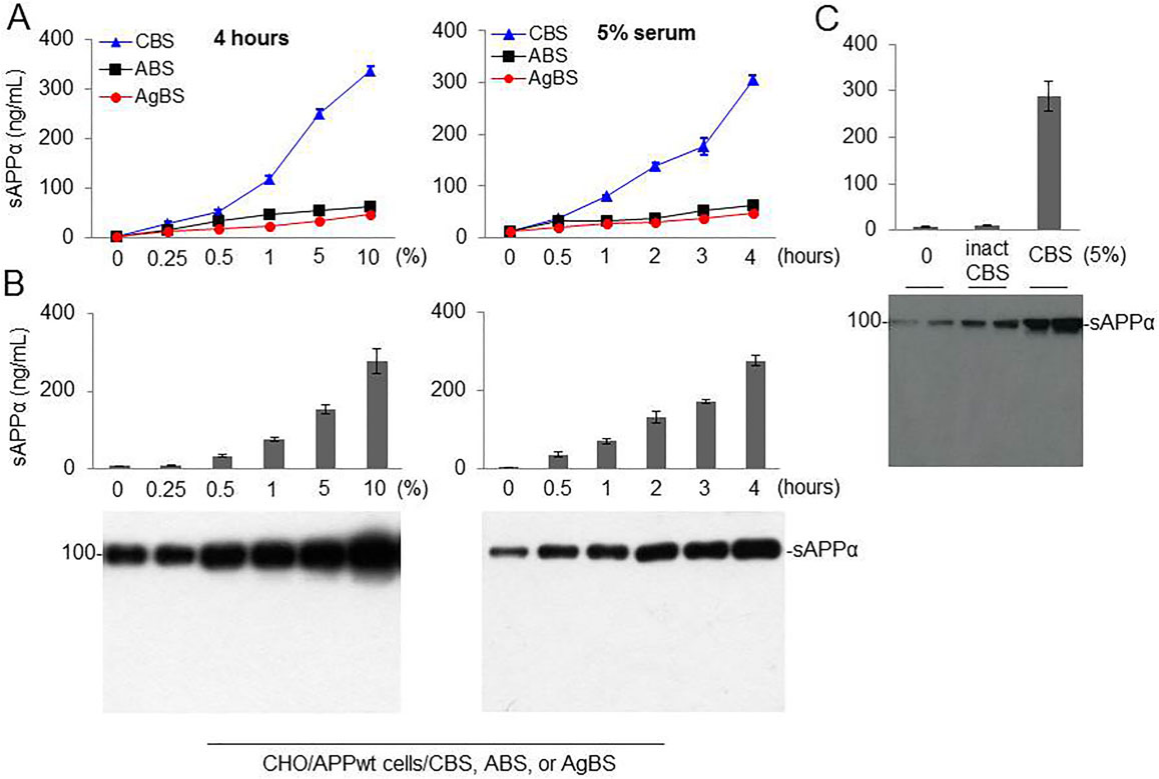
Cord blood serum (CBS), but not adult blood serum (ABS) or aged blood serum (AgBS), markedly promotes amyloid precursor protein (APP) α-cleavage in a time-, dose-, and temperature-dependent manner. (A) Chinese hamster ovary cells stably expressing wild-type human APP (CHO/APPwt) cells were treated with 0, 0.25, 0.5, 1, 5, and 10% CBS, ABS, or AgBS (left panel). In addition, CHO/APPwt cells were treated with 5% CBS, ABS, or AgBS between 0 h and 4 h as indicated (right panel). (B) CHO/APPwt cells were treated with CBS at different concentration (0% to 10%) for 4 h (left panel) or treated with 5% CBS for different time point (0 to 4 h), as indicated (right panel). Conditioned media were subjected to soluble amyloid precursor protein α (sAPPα) ELISA (Fig. 1A and B, top panel) and Western blot (WB) analyses (Fig. 1B, bottom panel) with 2B3 antibody (C) CHO/APPwt cells treated with heat inactivated serum (inact CBS) or CBS for 4 h. Conditioned media were subjected to sAPPα ELISA (Fig. 1C, top panel) and WB analyses (Fig. 1C, bottom panel) with 2B3 antibody. Data are presented as mean (±*SD*) of sAPPα produced (ng/mg or ng/mL) from 5 independent experiments in triplication. Human umbilical cord blood plasma (CBP) produced similar results (data not shown). APP α-secretase activity of pooled CBS or CBP or individualized CBS or CBP was similar (data not shown).

Our previous studies indicate that both single and multiple low-dose infusions of HUCBC as well as HUCBC-derived monocytes can significantly reduce β-amyloid plaques and cognitive impairments in AD mouse models. Having shown that HUCBC can reduce AD pathology, we next set out to determine whether human umbilical–derived CBS could also reduce β-amyloid pathology through alteration of APP processing. CHO/APPwt cells were treated with different concentrations (0% to 10%, 6 different doses) of CBS, ABS, or AgBS for 4 h (Fig. 1A and B, left panel). The conditioned media were collected and subjected to sAPPα ELISA, and also sAPPα WB analysis using 2B3 sAPPα-specific antibody. CBS dose-dependently promoted sAPPα levels with greater than that elicited by ABS and AgBS. Similarly, CHO/APPwt cells were treated with 5% CBS, ABS, or AgBS for 6 different time points (0 to 4 h, Fig. 1A and B, right panel). CBS time-dependently promoted sAPPα levels with greater than that elicited by ABS and AgBS. To see whether the factor present in the serum mediating α-secretase activity is proteinaceous in nature, we treated CHO/APPwt cells with heat inactivated serum (inact CBS) for 4 h. As expected, heat inactivation limited the sAPPα producing capacity of CBS, as shown by ELISA (Fig. 1C, upper panel) and WB (Fig. 1C, lower panel). Therefore, CBS possesses α-secretase, reflecting sAPPα level in a dose- and time-dependent fashion and the factor mediating this activity is heat-labile and most likely a protein. These results indicate that FBS also contains a heat sensitive α-secretase.

### CBS Mediates α-Cleavage of Neuron-specific APP_695_ Independent of ADAM Activity

**Fig. 2.**
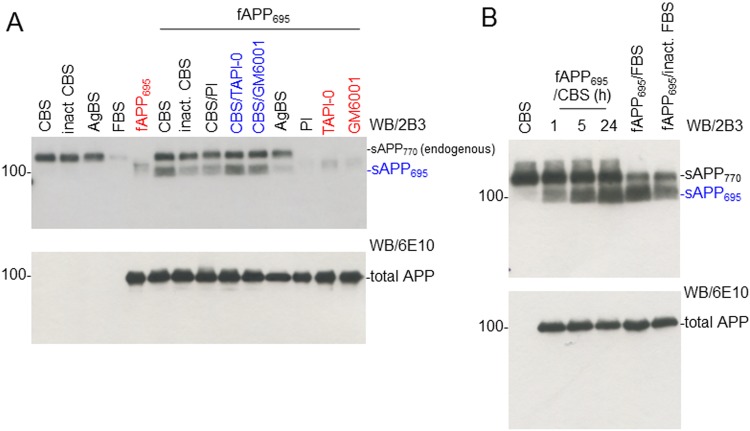
Cord blood serum (CBS) directly mediates α-cleavage of neuron-specific amyloid precursor protein (APP_695_), but this activity is not mediated by a disintegrin and metalloproteinase domain-containing protein (ADAM) and tumor necrosis factor-α converting enzyme (TACE). (A) Human recombinant full-length APP_695_ tagged with C-terminal MYC/DDK (fAPP_695_, OriGen, 100 ng) was incubated with 5% CBS, inact CBS, or aged blood serum (AgBS) at 37 °C for 5 h in the presence or absence of a protease inhibitor cocktail (PI, 1 X), TACE (ADAM17) inhibitor (TAPI-0, 1 µM), or ADAM inhibitor (GM6001, 1 µM). Lanes 1 to 4 represent CBS (1), inact CBS (2), AgBS (3), and fetal bovine serum (FBS; 4) sample only control without substrate fAPP_695_. Lane 5 represents fAPP_695_ (5) substrate only control without any serum sample. Lanes 6 to 11 represent substrate fAPP_695_ with CBS (6), inact CBS (7), CBS with PI (8), CBS with TAPI-0 (9), CBS with GM6001 (10), and AgBS (11). Lanes 12 to 14 represent (lanes 12 to 14; PI (12), TAPI-0 (13), and GM6001 (14) inhibitor and substrate control, respectively, without any serum sample. The reaction mixtures were subjected to soluble amyloid precursor protein α (sAPPα) Western blot (WB) analysis using 2B3 antibody (top panel) and total APP using 6E10 (an anti-Aβ_1-17_ antibody; lower panel). sAPP_770_ refers to the endogenous α-secretase cleavage product of CBS or AgBS, whereas sAPP_695_ refers to the α-secretase cleavage product of fAPP_695_. (**B**) 100 ng of fAPP_695_ was incubated with 5% CBS for 1, 5, or 24 h, or 5% FBS or inact. FBS for 24 h, and then subjected to sAPPα and total APP WB analysis using 2B3 (top panel) and 6E10 (lower panel), respectively. sAPP_770_ refers to the endogenous α-secretase cleavage product of CBS or AgBS, whereas sAPP_695_ refers to the α-secretase cleavage product of fAPP_695_.

Next, we tested whether the α-secretase in CBS is mediated by TACE (ADAM17) or ADAM. Human recombinant fAPP_695_ (100 ng)-tagged with C-terminal MYC/DDK was incubated with CBS, inactivated CBS (inact CBS), or AgBS at 37 °C for 5 h in the presence or absence of different inhibitors (PI cocktail [1X], TAPI-0 [1 µM], or ADAM inhibitor [GM6001, 1 µM]; Fig. 2A). The reaction mixtures were subjected to sAPPα WB analysis using 2B3 sAPPα-specific antibody (Fig. 2A, upper panel) and total APP analysis using 6E10 anti-Aβ_1-17_ antibody (Fig. 2A, lower panel). PI cocktail significantly limited CBS-derived α-secretase catalytic activity, as reflected by sAPP_695_ level, but this activity was not limited by TACE or ADAM inhibitors (Fig. 2A, upper panel). In addition, fAPP_695_ (100 ng) was incubated with 5% CBS, FBS, or inactivated CBS for 1, 5, or 24 h. CBS α-secretase increased the level of sAPP_695_ in a time-dependent manner, measured by 2B3 antibody (Fig. 2B, upper panel). As shown, the level of sAPP_770_ represents endogenous sAPPα.

### Removal of High- and Low-abundance Proteins Increases Activity of CBS α-secretase

**Fig. 3.**
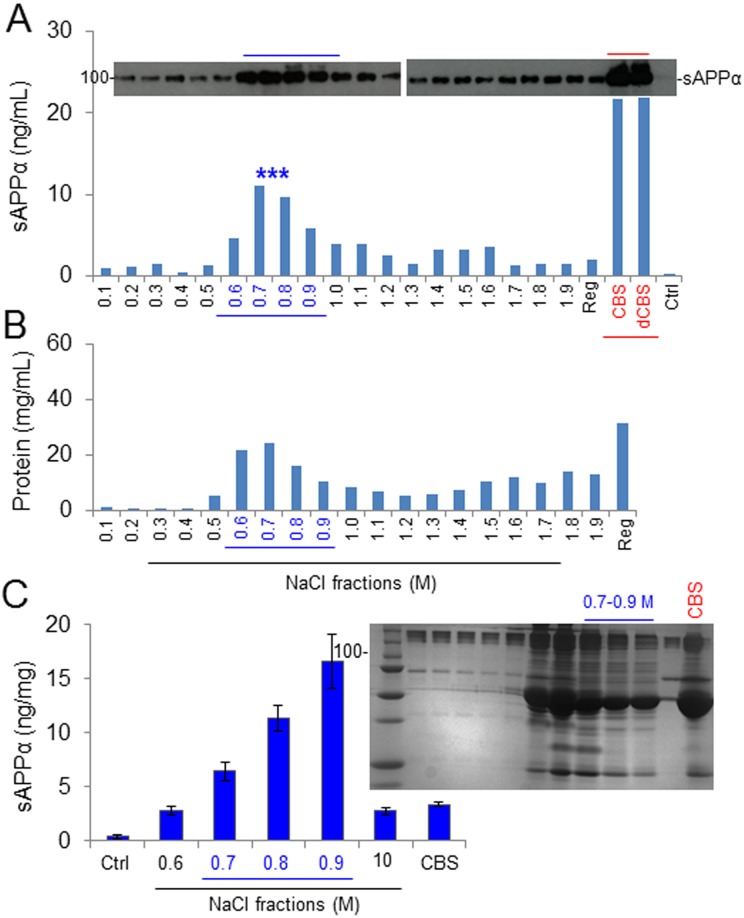
Fractionation of amyloid precursor protein (APP)-specific α-secretase activity in cord blood serum (CBS). To purify and eventually identify the α-secretase activity in CBS, the Econo-Pac Serum IgG Purification Kit (Bio-Rad, Philadelphia, PA, USA) was initially employed to remove highly abundant IgG. CBS was then desalted using Econo-Pac 10DG columns. The desalted serum was applied to DEAE Affi-Gel Blue columns to remove residual IgG and collect 20 additional protein fractions, by eluting with an increasing ionic strength gradient of NaCl buffer ranging from 0.1 M to 2.0 M. The remaining proteins on the column were eluted by the regeneration buffer included in the kit and collected as the regeneration fraction (Reg). (A) Chinese hamster ovary cells stably expressing wild-type human APP (CHO/APPwt) cells were cultured in 24-well plates and treated with 10 μL of each protein fraction for 2 h. Conditioned media were then collected and analyzed by soluble amyloid precursor protein α (sAPPα) Western blot (upper panel) and ELISA (lower panel). 10 μL CBS, desalted CBS, and phosphate-buffered saline (PBS; Ctrl) were included under the same cell culture conditions as positive and negative controls, respectively. Cell lysates were also prepared from each fraction-treated cell culture as an additional reference to evaluate sAPPα production levels (data not shown). (B) Protein concentration of each fraction. (C) CHO/APPwt cells were treated with the 0.6 to 1.0 M NaCl-eluted fractions from 10 different CBS lots, as well as whole CBS and PBS (Ctrl), for 2 h and the conditioned media were collected for sAPPα ELISA. The results were presented as mean (±*SD*) sAPPα produced (ng/mg protein). In addition, each protein fraction was subjected to sodium dodecyl sulfate polyacrylamide gel electrophoresis to assess total protein fractionation (C, right panel).

To purify and ultimately identify the target protein/complex mediating CBS α-secretase catalytic cleavage, 3-step chromatographic separation techniques were employed. Initially, removal of highly abundant immunoglobulins and desalting were performed using Bio-Rad Econo-Pac Serum IgG Purification Kit and 10DG columns. Desalted CBS was then applied to DEAE Affi-Gel Blue columns to completely remove IgGs and collect 20 additional protein fractions and eluted with increasing strengths of NaCl buffer. CHO/APPwt cells were treated with each fraction for 2 h to determine α-secretase by ELISA. In addition, unfractionated whole and desalted CBS positive control as well as PBS-negative control was used to treat cells. These sequential approaches significantly increased α-secretase, with the fractions showing the highest α-secretase catalytic activity, as reflected by sAPPα level eluting around 0.6 to 0.9 M NaCl concentrations (Fig. 3A). As shown in Fig. 3C, 0.7 to 0.9 M NaCl fractions from 10 CBS lots increased sAPPα levels at least with maximum 5-fold higher than whole CBS. In addition, the fractionated and whole CBS was run in SDS-polyacrylamide gel electrophoresis (PAGE), demonstrating numerous proteins remaining in each CBS fraction (Fig. 3C, right upper panel). Therefore, we selected the 0.8 M NaCl-eluted fraction for further purification. As shown in Fig. 3B, the level of total protein concentration is represented in mg/mL in CBS fraction.

### Further Purification of CBS α-Secretase Using Size-exclusion and Anion-exchange Chromatography

**Fig. 4.**
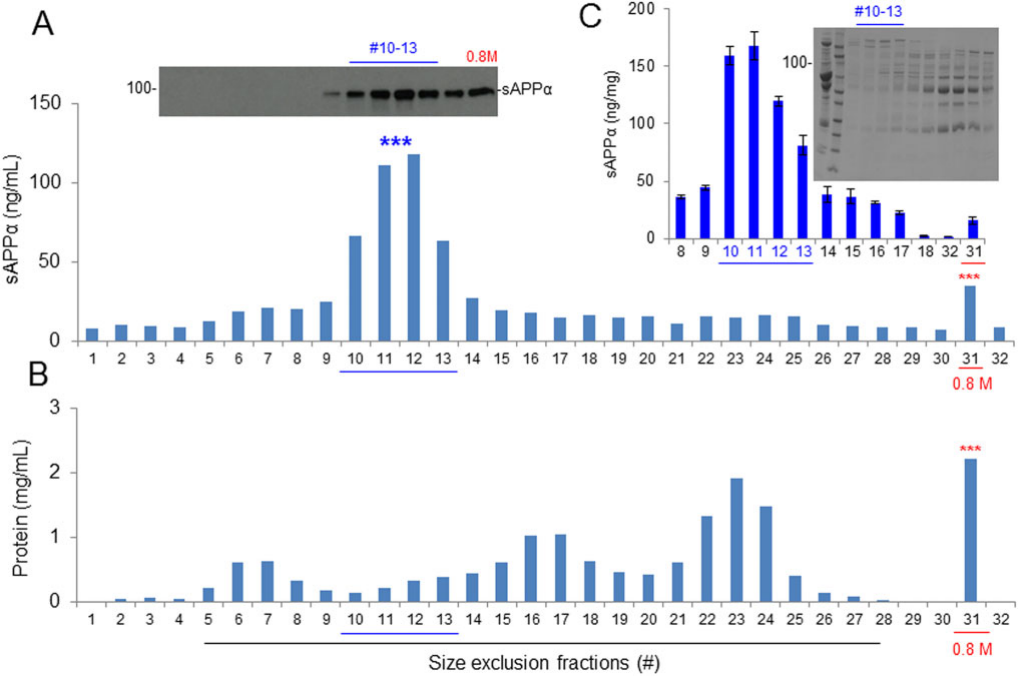
Protein size-exclusion chromatography by preparative-grade Superdex 200 column. The 0.8 M NaCl-eluted protein fraction of cord blood serum was further subjected to size-exclusion chromatography by analytic Superdex 200 column. Approximately 100 mg of protein from the 0.8 M NaCl fraction was applied to the column, and 48 fractions were eluted with phosphate-buffered saline (PBS). (A) Chinese hamster ovary cells stably expressing wild-type human APP (CHO/APPwt) cells were cultured in 24-well plates and treated with 40 μL of each protein fraction for 2 h. The conditioned media were collected and analyzed by soluble amyloid precursor protein α (sAPPα) Western blot (upper panel) and ELISA (lower panel). In parallel, the 0.8 M NaCl-eluted fraction (#31) and PBS (#32) were included under the same cell culture conditions as positive and negative controls, respectively. Cell lysates were also prepared from each fraction-treated cell culture as an additional reference to evaluate sAPPα levels. (B) Protein concentration of each size fraction. (C) CHO/APPwt cells were treated with #8 to 18 size fractions prepared from 3 independent experiments, as well as the original 0.8 M NaCl fraction (#31) and PBS (#32), and then conditioned media were collected and analyzed by sAPPα ELISA. The results were presented as mean (±*SD*) sAPPα produced (ng/mg protein). In addition, each size fraction was subjected to sodium dodecyl sulfate polyacrylamide gel electrophoresis analysis to assess total protein fractionation (C, right panel).

The 0.8 M NaCl-eluted fraction of CBS was subjected to size-exclusion chromatography using Superdex 200 prep grade columns (XK 16/40, GE Healthcare, PA, USA) packed with cross-linked agarose and dextran. The mobile phase was 20 mg/mL acetone in distilled water, and the detection was performed at UV280 nm. Approximately 100 mg of protein from the 0.8 M NaCl fraction was applied to the column, and 48 fractions were eluted with PBS. The catalytic activity of α-secretase was greatly enhanced, as tested on CHO/APPwt cells by measuring sAPPα production. Fractions #11 and 12 produced sAPPα > 5-fold higher compared with the original 0.8 M NaCl-eluted fraction as well as all other fractions, as determined by WB (upper panel) and ELISA (Fig. 4A, lower panel). To confirm the enhancement of α-secretase, we determined the sAPPα in fractions #8 to 18 along with the original 0.8 M NaCl-eluted fraction from 3 different CBS lots. Fractions #10 to 13 showed α-secretase activity 15-fold more than the original 0.8 M NaCl-eluted fraction, as measured by ELISA (Fig. 4C). As determined by SDS-PAGE, the molecular mass of the #10 to 13 fractions was 177 to 275 kDa (Fig. 4C).

**Fig. 5.**
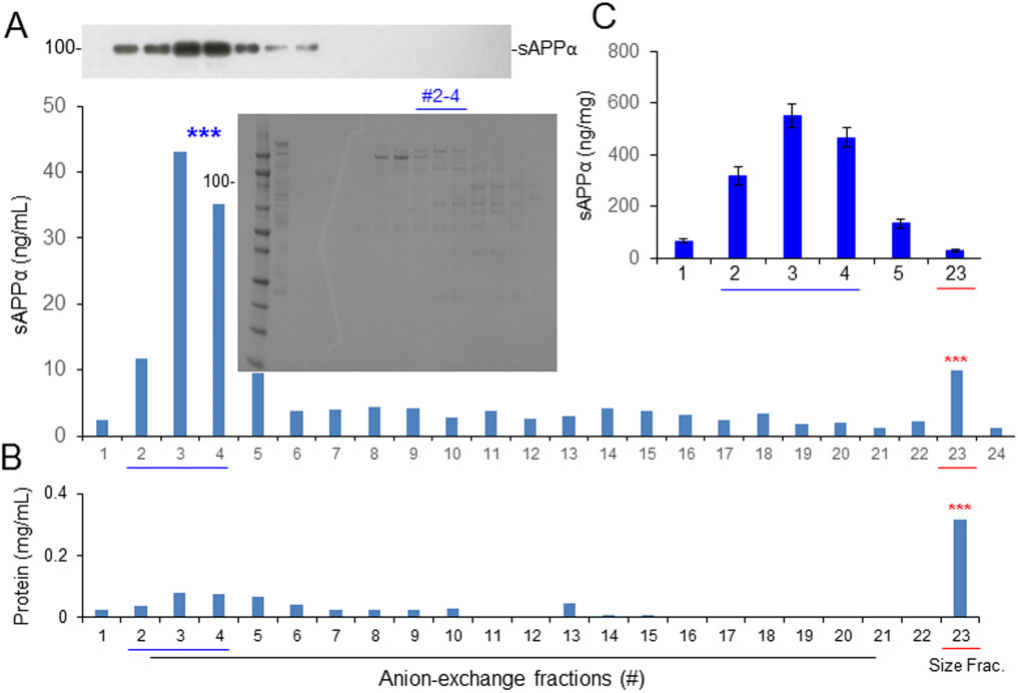
Further fractionation by anion-exchange chromatography. The size-exclusion fractions containing the highest amyloid precursor protein (APP) α-secretase activities (#10 to 13) were further subjected to anion-exchange chromatography using Q-Sepharose columns. Approximately 10 mg of protein from the size-exclusion fraction(s) was applied to the column, and 82 fractions were eluted with buffer containing 50 mM Tris, 500 mM NaCl, pH 7.6. (A) Chinese hamster ovary cells stably expressing wild-type human APP (CHO/APPwt) cells were treated with 40 μL of each protein fraction for 2 h and conditioned media and cell lysates were analyzed by soluble amyloid precursor protein α (sAPPα) Western blot (upper panel) and ELISA (lower panel). The original size exclusion–eluted fraction (#23) and phosphate-buffered saline (#24) were included as positive and negative controls respectively. In addition, sodium dodecyl sulfate polyacrylamide gel electrophoresis of #2 to 4 anion-exchange fractions showed the presence of multiple proteins (middle panel). (B) Protein concentration of each fraction. (C) CHO/APPwt cells were treated with the #1 to 4 anion-exchange fractions prepared from 3 independent experiments, and then conditioned media were subjected to sAPPα ELISA. The results are presented as mean (±*SD*) of sAPPα produced (ng/mg protein). The original size exclusion–eluted fraction (#23) was included as a positive control. Combined fractions #2 to 4, referred to as αCBSF, were used for further analysis.

To examine the charge of α-secretase protein/complex in CBS, the size-exclusion fractions containing the highest α-secretase catalytic activities, as reflected by sAPPα levels (#10 to 13), were further subjected to anion-exchange chromatography using Q-Sepharose columns. Proteins from the size-exclusion fractions were applied to the column and 82 fractions were eluted with 0.5 M NaCl. Fractions #53 to 56 showed > 8-fold higher α-secretase catalytic activity, as reflected by sAPPα level than the original size-exclusion fraction, as determined in CHO/APPwt cells by WB and ELISA (fractions #2 to 4 in Fig. 5A, upper and lower panels, respectively). To further compare the enzymatic activity of CBS samples before and after anion-exchange chromatography, we collected fractions #1 to 5 and #23 from 3 different samples and determined the sAPPα level in each indirect measurement of CBS α-secretase activity. Combined fractions #2 to 4 produced sAPPα > 50-fold higher than the original eluted (#23) fraction, as measured by ELISA (Fig. 5C). Combined fractions #2 to 4, referred to hereafter as αCBSF, were therefore used for further analysis.

### αCBSF Promotes Nonamyloidogenic APP Processing

**Fig. 6.**
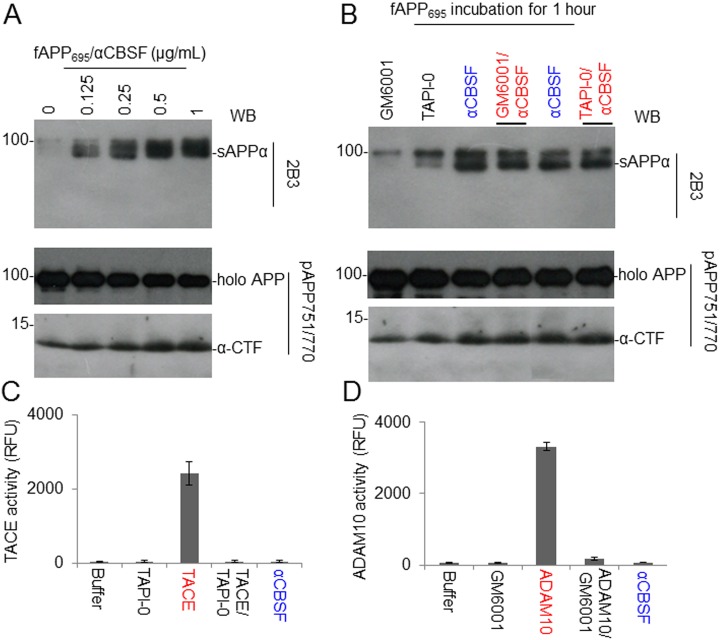
Cord blood serum–specific fraction with enhanced α-secretase catalytic activity (αCBSF) directly mediates α-cleavage of neuron-specific amyloid precursor protein (APP_695_), but this activity is not mediated by a disintegrin and metalloproteinase domain–containing protein (ADAM) or tumor necrosis factor-α converting enzyme (TACE). (A) Human recombinant full-length APP_695_ (fAPP_695_, 100 ng) was incubated with 0, 0.125, 0.25, 0.5, or 1 µg of αCBSF at 37 °C for 2 h. The reaction mixtures were subjected to soluble amyloid precursor protein α (sAPPα) Western blot (WB) analysis using 2B3 (top panel) and holo APP and α-C-terminal fragment (α-CTF) analysis using pAPP751/770 antibody (lower panel). (B) fAPP_695_ (100 ng) was incubated with 0.125 µg of αCBSF in the absence or presence of ADAM (GM6001, 1 µM) or TACE inhibitor (TAPI-0, 1 µM) for 1 h and then subjected to sAPPα, holo APP, and α-CTF WB analysis using 2B3 (top panel) and pAPP751/770 (lower panel). α-CTF of APP was further confirmed by an additional WB using an antibody specifically against Aβ_17-26_ (4G8). In addition, incubating human recombinant fAPP_751_ with αCBSF produces similar results (data not shown). (C) The tumor necrosis factor-α converting enzyme (TACE or ADAM17) activity of αCBSF was measured by TACE cleavage activity kit. TACE (25 μg/mL) secretase in the presence or absence of TACE inhibitor (TAPI-0, 1 µM) was included as positive control. (D) In parallel, the ADAM10 activity of the αCBSF was measured by ADAM10 cleavage activity kit. ADAM10 (50 μg/mL) secretase, in the presence or absence of ADAM inhibitor (GM6001, 1 μM), was included as positive control. TACE (ADAM17), and ADAM10 cleavage activities were determined for 1 h and expressed as relative fluorescence units. These results are presented as mean (±*SD*) of 3 independent experiments with triplicates for each condition.

Human recombinant fAPP_695_ was incubated with 5 different concentrations (0% to 1%) of αCBSF at 37 °C for 2 h. The reaction mixtures were subjected to sAPPα WB analysis using 2B3 antibody as well as fAPP and α-CTF analyses using pAPP751/770 antibody (an anti-APP C-terminal antibody; Fig. 6A). This analysis showed that αCBSF increases sAPPα as well as α-CTF fragments and decreases (full-length) holo APP with increasing doses.

In order to confirm that αCBSF mediates novel α-secretase independent of TACE or ADAM, fAPP_695_ was incubated with αCBSF in the absence or presence of ADAM (GM6001, 1 µM) or TAPI-0 (1 µM) for 1 h. αCBSF treatment alone increased the levels of APP-processing fragments such as sAPPα and α-CTF and decreased the level of holo APP as determined by WB, whose effects did not alter significantly by GM6001 and TAPI-0 combined treatment with αCBSF (Fig. 6B). In addition, TACE and ADAM10 enzymatic activities of αCBSF were measured by TACE and ADAM10 cleavage activity kits. TACE (25 μg/mL) in the presence or absence of TAPI-0 (1 µM) and ADAM10 (50 μg/mL) in the presence or absence of ADAM inhibitor (GM6001, 1 μM) were included as positive controls. Results suggest that αCBSF has very little TACE or ADAM10 activity (Fig. 6C and D).

### Immunoprecipitation of fAPP_695_/CBS Specifically Limits APP α-secretase Cleavage

**Fig. 7.**
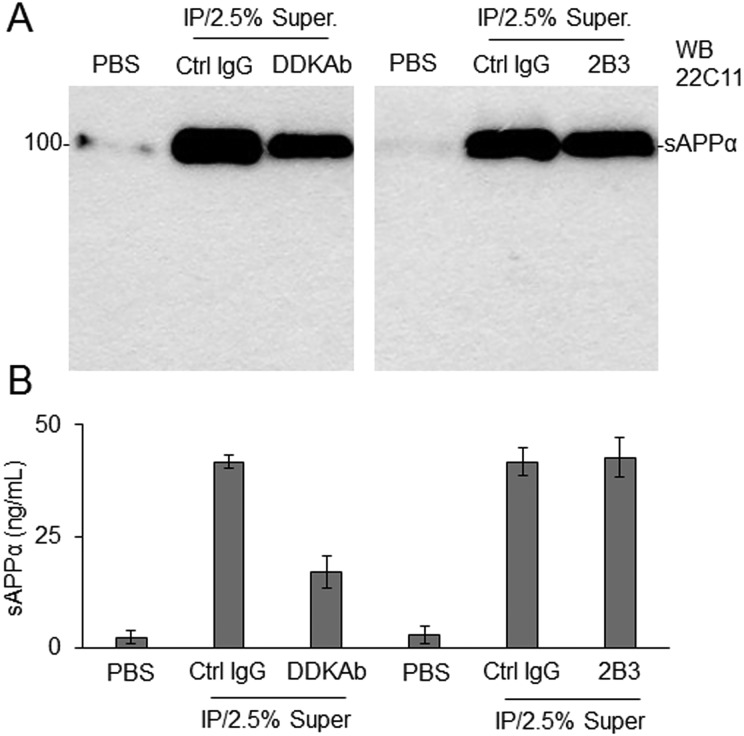
Immunoprecipitation of full-length amyloid precursor protein (APP_695_)/cord blood serum–specific fraction with enhanced α-secretase catalytic activity (αCBSF) specifically limits APP α-secretase activity of αCBSF. To determine whether immunoprecipitation could limit the ability of αCBSF to promote APP α-cleavage, we incubated 100 ng of fAPP_695_ with 0.125 μg of αCBSF at 4 °C for overnight and then immunoprecipitated (IP) the soluble amyloid precursor protein α (sAPPα)/αCBSF immune complex using an anti-DDK antibody (DDKAb), an sAPPα-specific antibody (2B3), or nonspecific IgG. Chinese hamster ovary cells stably expressing wild-type human APP cells were treated with the supernatants (Super.) from each immune complex, or phosphate-buffered saline as control, in the fetal bovine serum–free condition. Two hours after treatment, conditioned media were collected and analyzed by sAPPα Western blot (WB) using anti-N-terminal APP antibody (22C11, A) and sAPPα ELISA (B). For Panel (B), the results were presented as mean (±*SD*) of sAPPα production (ng/mL) in the conditioned media from 3 independent experiments with triplicates for each condition. There was no notable or significant difference in sAPPα production elicited by 0.5% supernatant from αCBSF immunoprecipitated with control IgG (Ctrl) and 2.5% (equivalent to 0.5% Super.) of αCBSF alone, as determined by sAPPα WB and ELISA analysis (data not shown).

To determine whether immunoprecipitation could specifically limit α-secretase of αCBSF, 100 ng of fAPP_695_ was incubated with 0.125 μg of αCBSF at 37 °C for 1 h, and the sAPPα/αCBSF immune complex was immunoprecipitated using anti-DDK antibody, 2B3, or nonspecific IgG. CHO/APPwt cells were treated in the FBS free condition for 2 h with the supernatants collected from the immune complex or PBS as reference control, and then conditioned media were analyzed by sAPPα WB using anti-N-terminal APP antibody (22C11; Fig. 7A) and sAPPα ELISA (Fig. 7B) to determine α-secretase in CBS. Immunoprecipitation of the sAPPα/αCBSF with anti-DDK antibody significantly reduced α-secretase activity of αCBSF, indicating that immunoprecipitation limits the CBS-mediated APP α-secretase cleavage. In contrast, immunoprecipitation of sAPPα/αCBSF with 2B3 did not reduce CBS α-secretase, indicating that αCBSF does not form an immune complex with sAPPα. In addition, there was no notable or significant difference in sAPPα production elicited by 0.5% supernatant from αCBSF IP with control IgG and 2.5% (equivalent to 0.5% Super) αCBSF alone (data not shown).

### αCBSF Reduces β-cleavage, Promotes α-cleavage of APP, and Stabilizes Tau Phosphorylation in 3xTg-AD Mice

**Fig. 8.**
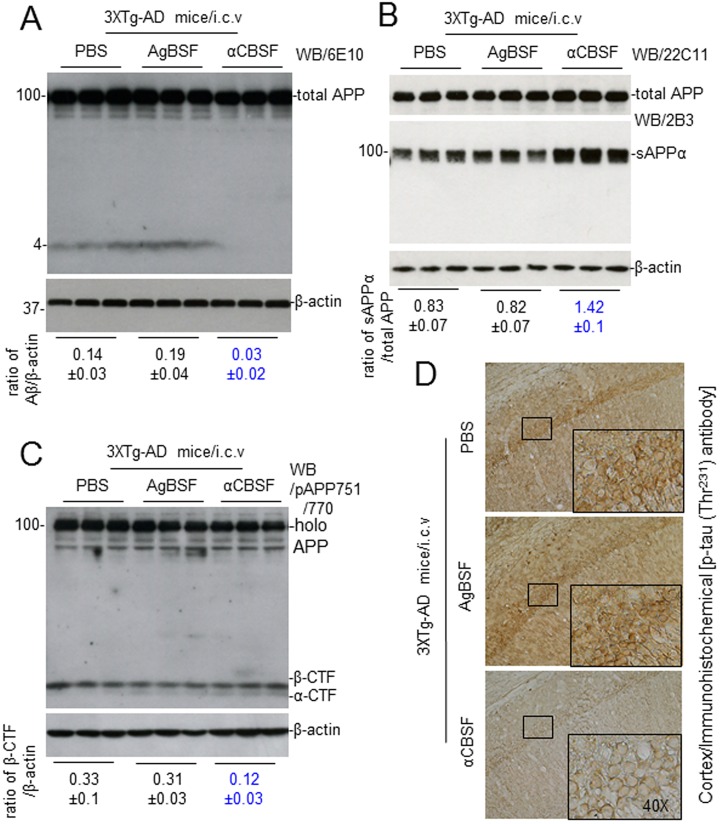
Cord blood serum–specific fraction with enhanced α-secretase catalytic activity (αCBSF) promotes amyloid precursor protein (APP) α-secretase processing in vivo. 3xTg-AD female mice at 4 mo of age were treated with αCBSF, aged blood serum fraction with enhanced α-secretase activity (0.5 μg/mouse; *n* = 6), or phosphate-buffered saline control (1 μL/mouse; *n* = 5 female) by i.c.v. injection and euthanized 72 h later. Mouse brain homogenates were then prepared from the right half of the brain (noninjection side). (A) Western blot (WB) analysis using Aβ_1-17_ antibody (6E10) shows total APP and Aβ species. (B) WB analysis using a soluble amyloid precursor protein α (sAPPα)-specific antibody (2B3) or anti-N-terminal APP antibody (22C11) shows sAPPα or total APP, respectively. (C) WB analysis using pAb751/770 shows full-length APP (holo APP) and 2 bands corresponding to β-carboxy terminal fragment and α-C-terminal fragment. (D) Mouse brain cortices from each treatment group were stained with anti-phospho-tau (p-tau [Thr^231^]) antibody. In addition, percentages (p-tau [Thr^231^] positive area/total area; mean ± *SD*) of anti-p-tau antibody positive cells were quantified by ImageJ (1.47v, NIH, USA) analysis (**P < 0.005; data not shown). WB data presented here are representative of results obtained from 5 to 6 female mice per group.

To test whether αCBSF suppresses β-site APP-cleaving enzyme 1 (BACE1)-mediated APP processing in vivo, 4-mo-old 3xTg-AD mice were treated αCBSF, AgBSF (0.5 μg/mouse), or PBS control (1 μL/mouse) with i.c.v. injections. After 72 h of treatment, in homogenates prepared from the right hemisphere (noninjection side), WB analysis using Aβ_1-17_ antibody (6E10) shows that αCBSF reduced Aβ (Fig. 8A) and β-CTF production (Fig. 8C), whereas enhancing sAPPα production (Fig. 8B) compared with AgBSF and PBS control. Compared with AgBSF and PBS, αCBSF also reduced tau (Thr^231^) phosphorylation in these mice (Fig. 8D).

### αCBSF Ameliorates β-amyloid Pathology in 5XFAD Mice

**Fig. 9.**
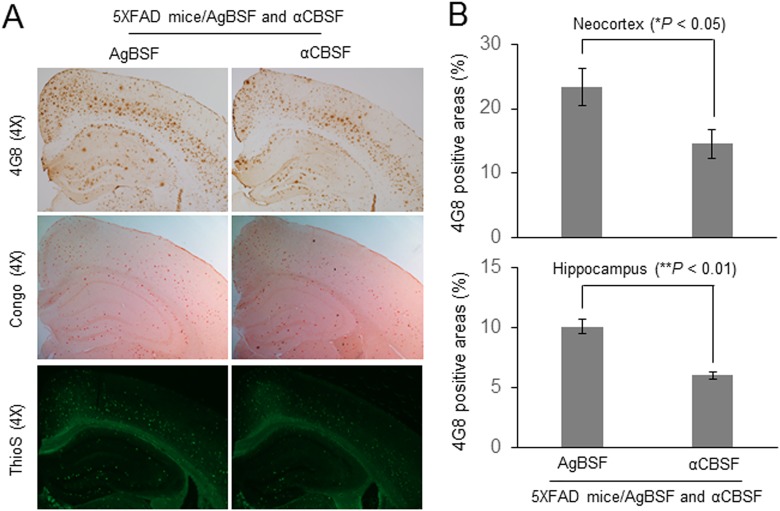
Cord blood serum–specific fraction with enhanced α-secretase catalytic activity (αCBSF) reduces β-amyloid plaques in 5XFAD mice. Five-month-old 5XFAD female mice were treated intraperitoneally with αCBSF (*n* = 5 to 7) and aged blood serum fraction with enhanced α-secretase activity (AgBSF; *n* = 5 to 6) *via* osmotic mini pump at 30 μg/mouse/day for 6 wk. (A) Mouse brain sections from each group were stained with 4G8, Congo red, and Thioflavin-S. (B) Percentages of 4G8 positive areas were quantified by ImageJ (1.47v, NIH, USA) analysis for neocortex and hippocampus, showing that αCBSF treatment significantly reduced plaque area compared with AgBSF treatment (*t*-test for independent samples; *P < 0.05, **P < 0.01).

To determine the effect of αCBSF on β-amyloid pathology, transgenic 5XFAD mice at the age of 5-month-old were continuously treated with αCBSF or AgBSF *via* i.p. osmotic mini pump for 6 wk. Immunohistochemical staining using 4G8 antibody showed that αCBSF treatment substantially decreases cortical and hippocampal β-amyloid plaques (Fig. 9A, upper panel) and reduces fibrillary Aβ species visualized by Thioflavin-S histochemical staining (Fig. 9A, lower panel) compared with AgBSF treatment. Moreover, the αCBSF-treated cohort also revealed less β-amyloid plaque pathology than the AgBSF-treated cohort, as determined by Congo red histochemical staining (Fig. 9A, middle panel). Quantitative analysis disclosed that αCBSF therapy significantly ameliorated β-amyloid pathology, as determined by 4G8 antibody staining in both neocortex and hippocampus regions compared with AgBSF treatment (Fig. 9B).

### Neuroprotective Effects of αCBSF

**Fig. 10.**
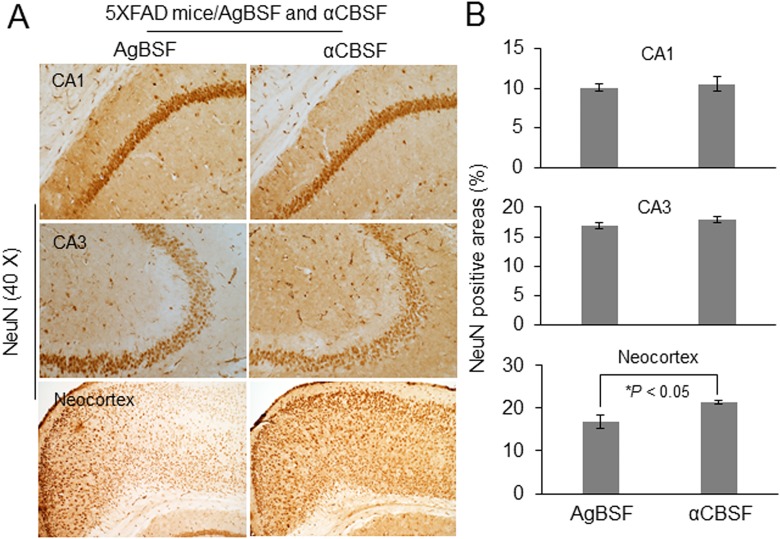
Neuroprotective effects of cord blood serum–specific fraction with enhanced α-secretase catalytic activity (αCBSF). 5XFAD mice at 5 mo of age were treated intraperitoneally with αCBSF (*n* = 7) or aged blood serum fraction with enhanced α-secretase activity (AgBSF; *n* = 6) *via* osmotic mini pump for 6 wk. (A) Mouse brain sections from αCBSF- and AgBSF-treated groups were stained with anti-NeuN antibody. (B) Quantification of NeuN positive cells in the CA1, CA3, and neocortex revealed that αCBSF treatment significantly increased NeuN-positive cells compared with AgBSF treatment in neocortex (*t*-test for independent samples; *P < 0.05).

5XFAD mice undergo neuronal loss in the neocortex and hippocampus that is associated with behavioral deficits. We examined whether continuous delivery of αCBSF by i.p. osmotic mini pump can elicit a neuroprotective effect in 5XFAD mice. Treatment with αCBSF partially prevented neuronal loss in the neocortex region compared with AgBSF treatment, as demonstrated by NeuN antibody immunohistochemical staining, thus indicating that αCBSF may confer neuroprotective ability for AD brain (Fig. 10A and B).

### αCBSF Improves Learning, Memory, and Cognitive Function in 5XFAD Mice

**Fig. 11.**
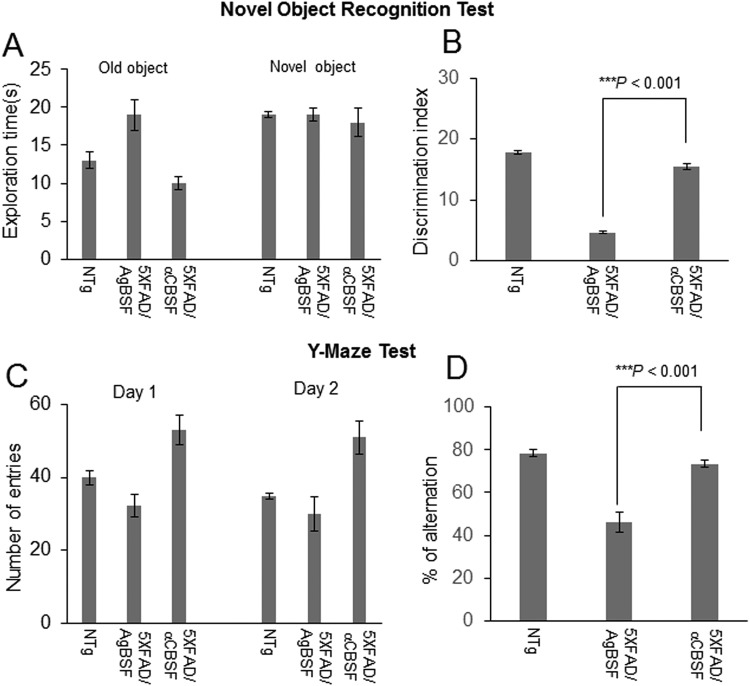
Cord blood serum–specific fraction with enhanced α-secretase catalytic activity (αCBSF) improves cognitive function in 5XFAD mice. Both male and female 5XFAD mice at 5 mo of age and age-matched nontransgenic wild-type (WT) controls (NTg) were treated with αCBSF and aged blood serum fraction with enhanced α-secretase activity (AgBSF; 30 μg/mouse/day) intraperitoneally by osmotic mini pump for 6 wk as described in the Methods and Materials section. Each treatment groups as well as nontransgenic WT controls (NTg) were subjected to Novel Object Recognition and Y-maze behavioral testing. (A) Times spent exploring old and novel objects during the test phase of novel object recognition was recorded for each treatment group. (B) Discrimination index, calculated as the frequency of exploring new object versus original objects, was significantly reduced in 5XFAD mice treated with AgBSF, but not in those treated with αCBSF, compared with NTg controls. (C) Total number of arm entries for Y-maze was recorded for each treatment group. (D) Percentage alternations was significantly reduced in 5XFAD mice treated with AgBSF, but not in those treated with αCBSF, compared with NTg controls. Significance level determined by analysis of variance for a total of *n* = 5, NTg mice; *n* = 6, αCBSF-treated 5XFAD mice; and *n* = 6 AgBSF-treated 5XFAD mice (***P < 0.001).

5XFAD mice received continuous treatment with αCBSF or AgBSF *via* i.p. osmotic mini pump for 6 wk and evaluated for cognitive function by novel object recognition and Y-maze tests during 4-5 wk of treatment. Novel object recognition test showed that αCBSF-treated 5XFAD mice spent more time with the novel *versus* old objects, whereas AgBSF-treated 5XFAD mice spent the same period of time with both novel and old objects (Fig. 11A). Thus, discrimination index (%) was enhanced by αCBSF compared with AgBSF treatment (Fig. 11B). Notably, improvement was complete because there was no significant difference (*P* > 0.05) from WT control mice (NTg). In addition, αCBSF treatment significantly increased the number of entries (Fig. 11C) and spontaneous alterations in 5XFAD mice compared with the AgBSF-treated cohort (Fig. 11D), as determined by Y-maze test, thus confirming that αCBSF treatment improved learning and working memory in this AD mouse model.

## Discussion

Recent progress in HUCBC therapy for different neurological diseases^26,27^ opened new opportunities for AD research^28^. We have previously found that multiple low-dose peripheral infusion of HUCBC reduced cerebral β-amyloid plaques, cerebral amyloid angiopathy, and astrocytosis, whereas these treatments improved cognitive impairments in the PSAPP AD mouse model^11^ and enhanced neurogenesis in the aged rat brain^29^. In a subsequent study, we have reported that HUCBC-derived monocytes reduced cerebral β-amyloid pathology and cognitive impairments^13^. In addition, we have revealed that HUCBC-derived monocyte more effectively removed Aβ by phagocytosis than the aged monocyte, whereas sAPPα enhanced Aβ phagocytosis by the aged monocyte by forming a complex with Aβ *via* the help of monocyte scavenger receptor^13^. In support of these findings, we demonstrated that overexpression of sAPPα significantly reduces both cerebral β-amyloid^6^ and tau pathology in crossing Tg-sAPPα with PSAPP mice^7^. Meanwhile, using a sophisticated parabiosis mouse model, Wyss-Coray and colleagues showed that blood serum from old mice reduces neurogenesis and impairs cognitive functions when administered into young mice^30^. Subsequently, several groups reversed age-related cognitive impairments in aged mice by infusing plasma from young into old mice^14–16^ as well as AD pathology^18^. More specifically, Wyss-Coray group have published several articles over the last few years^14,19^, showing the potential of young and/or umbilical cord plasma in ameliorating aged-associated cognitive impairments. In those experiments, they have either joined the young and aged mice through parabiosis or injected young and/or umbilical cord blood–derived plasma into aged mice *via* tail vein injection. Interestingly, only 3 to 4 injections within 3- to 4-wk period of time improved cognitive impairments in those experiments. These results encouraged us to determine whether human CBS could effectively reduce AD pathology in vitro (i.e., cell culture and cell-free systems) as well as in vivo (i.e., 3xTg-AD and 5XFAD mouse models) by enhancing sAPPα production.

Our preliminary findings indicate that CBS possesses α-secretase-like enzyme in cell culture and cell-free systems. In CHO/APPwt cells, CBS produces greater amount of sAPPα compared with ABS and AgBS in a concentration- and time-dependent manner (Fig. 1A and B). Since α-secretase is proteinaceous and heat-labile, we hypothesized that α-secretase-like enzyme displayed by CBS is also inactivated by heat treatment. As expected, heat inactivation significantly limited the sAPPα-producing capacity of CBS (Fig. 1C). These results suggest that α-secretase-like enzyme of CBS is most likely a single complex protein that interacts with and cleaves APP. Subsequent study indicated that CBS mediates α-secretase cleavage of neuron-specific APP_695_ in a cell-free system, further suggesting that this activity is mediated by an endogenous enzyme (Fig. 2A and B).

To purify, characterize, and ultimately identify this α-secretase-like content in CBS, we employed 3-step affinity column, size-exclusion, and anion-exchange chromatography techniques in a sequential manner (Fig. 3–5). These sequential purification steps enhanced the catalytic activity more than 3,000-fold compared with original CBS. The fractions containing highest α-secretase catalytic activity, as reflected by sAPPα level, were combined and termed as “αCBSF” for the further study. SDS-PAGE analysis of the fractions from size-exclusion and anion-exchange chromatography yielding the highest α-secretase indicated size of our unknown enzyme could be around 177 to 275 kDa (Figs. 4 and 5). It is not easy for a 177- to 275-kDa protein to cross the blood–brain barrier through i.p. mini pump administration without any inhibition. We do not believe the protein is larger than 275 kDa based on the markers in for our gels. However, the SDS-PAGE also showed some low-molecular-weight compounds that cannot be ruled out as well, which warrant further investigation. Interestingly, TAPI (ADAM17) and GM6001 (ADAM) inhibitors did not alter α-secretase in CBS, indicating that the enzyme is not TACE or ADAM, whereas the activity was dramatically reduced by PI/cocktail, confirming that the activity is mediated by a protease (Figs. 2 and 6). Moreover, immunoprecipitation of αCBSF with 2B3 antibody (anti-C-terminal of sAPPα) showed significant reduction in sAPPα levels, indicating that α-secretase-like enzyme αCBSF physically interacts with sAPPα (Fig. 7).

Previously, we and others have shown that sAPPα reduces β-amyloid pathology *via* inhibition of BACE1^6^. In a recent article, we have shown that sAPPα decreases tau phosphorylation *via* BACE1 inhibition and GSK-3β-mediated inhibitory phosphorylation^7^. This study prompted us to investigate the functional efficacy of fractionated CBS (αCBSF) in 5XFAD and 3xTg-AD mouse models. We have shown that αCBSF significantly reduced Aβ and tau phosphorylation (p-tau-Thr^231^) in 3xTg-AD mice, whereas αCBSF enhanced α-secretase cleavage products (i.e., sAPPα and α-CTF), indicating that α-secretase-like content in CBS promotes APP nonamyloidogenic processing in vivo (Fig. 8B and C). In 5XFAD mice with aggressive β-amyloid deposition and plaque formation, αCBSF reduced β-amyloid plaque pathology in both neocortex and hippocampus regions, and reduced neural loss in the neocortex region, compared with AgBSF-treated mouse brains (Fig. 9 and 10). By carrying out sequential fractionation, we markedly enhanced CBS-derived α-secretase (termed αCBSF) and infused into 5XFAD mice *via* osmotic mini pump over the period of 6 wk. Behavioral analyses in 5XFAD mice indicate that αCBS-treated mice showed improved episodic memory, as determined by novel object recognition test (Fig. 11A and B), as well as spatial working memory, as determined by Y-maze test (Fig. 11C and D), compared with AgBSF treatment. Our work is in line with the work of Villeda et al.^14^ and Castellano et al.^19^, where improvement of performance in cognitive impairment was found in aged mice treated with young plasma. Notwithstanding, we are not quite sure how CBS fraction (αCBSF) ameliorates β-amyloid pathology and cognitive functioning in 5XFAD and tau pathology in 3xTg-AD mouse model. The effect we observe may or may not be from CBS α-secretase-like enzyme. One of the plausible explanations for this effect may be a direct action from CBS α-secretase-like enzyme or could be an indirect effect through peripheral sink hypothesis which demand further investigation. Although we do not know the exact molecular mechanism, however, we believe that human cord blood–derived serum and/or plasma protein functions as a master regulator of several genes involved in the proliferation of cells, and blood vessels that might reduce neuroinflammation, Aβ, and improve synaptic plasticity by affecting multiple pathways. Overall, our results show beneficial effects of αCBSF in ameliorating β-amyloid pathology and cognitive functioning in 5XFAD and reducing tau phosphorylation in 3xTg-AD mouse models.

It is well known that members of the membrane-bound zinc-dependent metalloproteinase ADAM family are α-secretase enzymes that cleave APP for the nonamyloidogenic pathway. In particular, 3 different members of this family, ADAM9, ADAM10, and ADAM17, possess APP α-secretase activity^31^. The ADAM family constitutes a large family of multidomain membrane proteins that have cysteine-rich, disintegrin, and zinc metalloprotease domains in their ectodomain^32^. The main function of ADAM family is to shed the ectodomain of different membrane proteins and has growth factors-like function *via* intracellular signaling cascade. However, it should be noted that numerous other substrates also have been linked to this ADAM family. These functions of ADAM family either protect against AD or promote AD pathogenesis *via* activation of different cytokines. One of the enzymes, ADAM17, is also known as TACE, responsible for secreting the main pro-inflammatory cytokine, TNFα^33^. Thus, TACE (ADAM17) is a therapeutic target for multiple diseases. Additionally, both ADAM10 and ADAM17 cleave various other membrane proteins and promote tumor in the cell^34^. ADAM10, in particular, cleaves many different kinds of transmembrane proteins in the vascular system, including the platelet-activating collagen receptor glycoprotein VI^35,36^, and endothelial proteins, including transmembrane chemokines (i.e., CX3CL1 and CXCL16)^37^. These 2 chemokines are known for angiogenesis, inflammation, and immune cell recruitment^38,39^. Likewise, ADAM9 cleaves and releases a number of molecules with important roles in tumorigenesis and angiogenesis. Taken together, whereas the known α-secretase enzymes, mainly ADAM10 and ADAM17 (TACE) and in some degree ADAM9, are involved in APP α-secretase cleavage, they are not APP-specific and cleave various substrates associated with inflammation, tumor formation, and progression. Thus, whereas AD is the only pathology in which an increased α-secretase activity has been proposed to be favorable, the nonspecific nature of the known α-secretases has made this strategy for AD treatment thus far unsuitable^40^.

In sum, our study has presumably discovered an umbilical cord blood–derived α-secretase that is independent of TACE or ADAM, thus making it a suitable candidate for the further study as a therapeutic target for AD treatment. This α-secretase-like enzyme activity either directly or indirectly activates α-secretase or produces sAPPα in cell culture and AD animal models. In addition, we believe this α-secretase appears to be mediated by novel enzymes residing within the sera which decline with age. We expect that our study using fractionation, chromatographic separation, and mass-spectrometry (MS) techniques would identify the target enzyme as well as other interacting partners from CBS. However, identification of a target protein or enzyme with a particular function from a complex mixture of serum is a challenging task due to multiple factors including the high complexity and wide dynamic range of proteins as well as the presence of contaminating proteins of high abundance. Despite this, here we show that our purification techniques significantly enhanced the α-secretase of CBS. Further, MS-based sophisticated purification techniques will completely purify, identify, and characterize the factor mediating this α-secretase in CBS.
