# Expression of Concern

**DOI:** 10.1177/09636897221120609

**Published:** 2022-08-08

SAGE hereby issues an expression of concern for the following research article:

Habib A, Hou H, Mori T, et al. Human Umbilical Cord Blood Serum–derived α-Secretase: Functional Testing in Alzheimer's Disease Mouse Models. *Cell Transplantation*. March 2018:438-455. doi:10.1177/0963689718759473

A reader posted their concerns about images in this article on Pub Peer indicating a possible splice in Figure 6B α-CTF panel between lanes 4 and 5. The corresponding author did not respond to publisher requests for comment.

The remaining authors noted that the corresponding author has access to all of the data underlying the findings in this article and were unable to comment on the concerns raised.

The Editors and SAGE strive to uphold the very highest standards of publication ethics and are committed to supporting the high standards of integrity of *Cell Transplantation*. Authors, reviewers, editors and interested readers should consult the ethics section of SAGE and the Committee on Publication Ethics (COPE) website for guidelines on publication ethics.

We were unable to reach the following authors: Tian J, Zeng J, Fan S, Tan, J.

